# Up-to-Date Tailored Systemic Treatment in Pancreatic Ductal Adenocarcinoma

**DOI:** 10.1155/2019/7135437

**Published:** 2019-09-05

**Authors:** Caroline Y. K. Fong, Emma Burke, David Cunningham, Naureen Starling

**Affiliations:** The Royal Marsden NHS Foundation Trust, Downs Road, Sutton SM2 5PT, UK

## Abstract

Despite intensive research efforts, pancreatic ductal adenocarcinoma is still regarded as an aggressive and life-limiting malignancy. Combination chemotherapy regimens that underpin the current treatment approach in the advanced setting have led to incremental survival gains in recent years but have failed to confer patients with a median overall survival that exceeds 12 months from diagnosis. Research has since focussed on understanding the role and interplay between various components of the desmoplastic stroma and tumour microenvironment, in addition to developing targeted therapies based on molecular features to improve the prognosis associated with this malignancy. This review will summarise the available systemic treatment options and discuss potential methods to refine the resolution of patient selection to enhance responses to currently available therapies. Furthermore, it will explore newer approaches anticipated to come to the fore of future clinical practice, such as agents targeting the DNA damage response and tumour microenvironment as well as immunotherapy-based combinations.

## 1. Introduction

Pancreatic ductal adenocarcinoma (PDAC) is the 14^th^ most common malignancy worldwide, with a global incidence of 458,918 cases in 2018 [1]. In comparison, 432,232 deaths were attributed to PDAC, making it the seventh most common cause of cancer-related death worldwide [1].

At present, surgical resection is the sole curative treatment modality but is relevant to only 15% of patients presenting with a new diagnosis of PDAC who have resectable disease. Even so, current data indicates that patients who undergo curative resection followed by adjuvant chemotherapy have a limited prognosis consisting of a median overall survival (OS) ranging from 28 to 54 months [2–4] and that patients in the United Kingdom have a five-year survival rate of 3.3%, regardless of stage at diagnosis [5]. Treatment within the palliative paradigm consists of chemotherapy, with the most intensive combination achieving a modest median OS of 11.1 months [6].

The poor prognosis associated with PDAC is a culmination of vague symptomatology leading to late presentation, a complex mutational landscape, and a dense desmoplastic stroma with an immunosuppressive tumour microenvironment (TME) that collectively pose challenges in developing and delivering effective systemic treatments. By 2030, PDAC is projected to become the second leading cause of cancer deaths in America, second only to lung cancer [7]. This forecasted statistic reflects the stagnation of progress in PDAC management relative to other cancers despite intense research efforts over recent years and highlights the urgent need for novel approaches that can provide patients with clinically meaningful benefit. Here, we will outline the current standard-of-care in the advanced disease setting and discuss emerging treatment strategies approaching clinical practice.

## 2. Current Approach to Systemic Therapy

For thirty years, the cornerstone of systemic treatment for metastatic PDAC consisted of 5-fluorouracil (5-FU). This was surpassed in 1997, when patients randomised to receive gemcitabine monotherapy demonstrated an improvement in clinical benefit response, a composite measure consisting of pain, performance status, and weight, in comparison to patients treated with 5-FU (23.8% vs. 4.8%, *p* = 0.002) [8] (Table 1). Additionally, the gemcitabine group demonstrated a small survival advantage over patients who received 5-FU (median OS 5.65 vs. 4.41 months, *p* = 0.002).

Clinical trials examining the efficacy of gemcitabine-containing doublet regimens with a second chemotherapeutic agent or targeted therapies were largely negative [9–12]. There are notable exceptions. Although a phase III randomised trial comparing gemcitabine and erlotinib against gemcitabine alone showed a statistically significant improvement in both progression-free survival (PFS) and OS in the combination arm, this failed to translate clinically as it amounted to an absolute difference of 6 and 10 days, respectively [13]. Interestingly, a subset of patients with grade ≥ 2 skin rash obtained a more significant survival benefit compared to patients with milder or no skin toxicity (10.5 months vs. 5.8 months vs. 5.3 months, HR 0.74, *p* = 0.037). Nevertheless, this combination is rarely utilised in clinical practice as its potential benefit is limited to a small proportion of patients. A randomised controlled trial examining the efficacy of gemcitabine and capecitabine against gemcitabine monotherapy demonstrated statistically significant improved response rates (19.1% vs. 12.4%, *p* = 0.34) and median PFS (5.3 vs. 3.8 months, HR 0.78, *p* = 0.004), with a trend towards improved median OS (7.1 vs. 6.2 months, HR 0.86, *p* = 0.08) [14]. A meta-analysis of these results amalgamated with data from two further studies detected a survival benefit associated with gemcitabine and capecitabine over monotherapy (HR 0.86; 95% CI, 0.75 to 0.98; *p* = 0.02), providing support for the use of this regimen in treatment-naïve advanced PDAC patients [14].

Two further chemotherapy combinations provide treatment options in the first-line setting for advanced PDAC in patients fit for combination therapy. The triplet combination of 5-FU, irinotecan, and oxaliplatin (FOLFIRINOX) was shown to improve median OS to 11.1 months, in comparison to 6.8 months in patients receiving gemcitabine (HR 0.57, *p* < 0.001), with a parallel improvement in quality of life at 6 months as assessed by the EORTC QLQ-C30 questionnaire (HR 0.47, *p* < 0.001) [6]. Separately, gemcitabine and nanoparticle albumin-bound paclitaxel (nab-paclitaxel) also extended median OS to 8.5 months, in comparison to 6.7 months with gemcitabine monotherapy (HR 0.72, *p* < 0.0001) in patients with metastatic disease (Table 1) [15].

At face value, FOLFIRINOX may be perceived as the superior regimen due to its longer median OS, albeit with a less tolerable safety profile. However, a direct comparison of outcomes between both trials cannot be made due to variations in study methodologies and patient characteristics. A systematic review and meta-analysis of 16 retrospective studies involving 3,813 patients comparing the effectiveness of FOLFIRINOX against gemcitabine/nab-paclitaxel showed that both regimens resulted in similar OS, PFS, overall response rates, and risks of death and disease progression with distinct toxicity profiles [16]. These findings reinforce the notion that both combinations are reasonable upfront treatment options for patients with a good performance status, and the choice of treatment should ultimately be individualised accordingly to patient's comorbidities and preferences. In patients with poor performance status, gemcitabine monotherapy can still be considered in patients treated with palliative intent.

Approximately 40% of patients eventually receive second-line systemic therapy [17] following gemcitabine-based first-line therapy. There is currently no prospective trial data to determine the optimal sequencing of FOLFIRINOX and gemcitabine/nab-paclitaxel, and these remain as treatment options in patients requiring second-line therapy provided they remain fit for these regimens. Oxaliplatin-based chemotherapy has been considered as standard-of-care in this setting after oxaliplatin, leucovorin, and 5-FU (OFF) demonstrated superior median OS in the CONKO-003 trial when compared to leucovorin and 5-FU (FF) (5.9 vs. 3.3 months, HR 0.66, *p* = 0.001) [18]. These findings were challenged by results from the PANCREOX trial, which showed that survival outcomes were inferior in patients randomised to receive modified FOLFOX6 (mFOLFOX6; 5-FU, leucovorin, and oxaliplatin) in comparison to patients in the 5-FU-leucovorin arm [19]. A higher incidence of grade ≥ 3 adverse events and adverse events leading to treatment discontinuation was observed in the mFOLFOX6 arm compared to the 5-FU-leucovorin arm (63% and 20.4% vs. 11% and 1.9%, respectively). Furthermore, a higher proportion of patients in the 5-FU-leucovorin arm received postprogression therapy in comparison to patients assigned to receive mFOLFOX6 (25% vs. 6.8%), possibly due to the poorer tolerability associated with the former combination. As the median PFS of both arms were similar (HR 1.00, *p* = 0.36), the discrepancy in OS could be attributed to the differences in postprogression therapy uptake [19]. More recently, long-term data from the NAPOLI-1 trial [20] has since confirmed that nanoliposomal irinotecan and 5-FU-leucovorin is associated with superior median OS when assessed against 5-FU-leucovorin [21], providing a further option for patients with gemcitabine-refractory disease who remain well enough for additional treatment (Table 1). Although therapeutic options for second-line therapy are available, the risk benefit of pursuing further systemic treatment should be carefully considered in view of the significant symptomatic burden and rapid clinical deterioration commonly seen in these patients at this juncture.

## 3. Improving Patient Selection to Maximise Treatment Efficacy

In contrast with other malignancies, targeted therapies do not feature in the treatment armamentarium due to a lack of a targetable driver mutation in this disease. Although over 90% of PDAC cases harbour activating *KRAS* mutations [22], thus providing an attractive therapeutic target, KRAS is deemed “undruggable” due to its relatively smooth 3D structure [23]. Efforts to target downstream components of the RAS pathway, such as mitogen/extracellular signal-regulated kinase (MEK), have also been unsuccessful in the clinical setting [24–26]. The epidermal growth factor receptor (EGFR) is overexpressed in 80% [27] of PDACs, which collectively with KRAS mutations may explain the lack of efficacy from anti-EGFR-targeted therapies [13, 28]. Given that there has been a paucity of novel therapeutic agents coming to the fore of PDAC management, research activity has also focussed on devising methods to refine patient selection for treatment based on individual patient molecular profiles. Several molecular characterisations for PDAC exist [29–33] using tissue from both patients with early and advanced disease, and current classifications have identified molecular features with prognostic and responsive associations. For example, the basal subtype is associated with chemoresistance and poor prognosis, whereas the classical subtype is associated with a more favourable outlook [29]. The COMPASS trial was designed as a prospective study to assess the feasibility of comprehensive real-time whole genome sequencing and RNA sequencing of advanced PDAC to identify predictive mutational and translational features to inform treatment selection [34]. In addition to demonstrating that prospective genomic profiling was feasible with a median turnaround time of 35 days, the investigators detected better objective responses to first-line chemotherapy in patients with *GATA6*-amplified classical PDAC RNA subtype in comparison to basal-like patients (*p* = 0.004), with the best PFS observed in patients who received modified FOLFIRINOX [34]. While this finding provides some prospective evidence in a nonrandomised cohort that the characterisation of RNA subtypes via molecular profiling may allow upfront identification of chemosensitivity to potentially guide treatment selection, it requires further assessment in a controlled trial to fully ascertain its role in the clinic.

Another promising method to enhance treatment selection is through the use of patient-derived organoids (PDOs) to facilitate *in vitro* drug sensitivity testing [35]. PDOs are cultured from LRG5^+^ stem cells that can be isolated from a number of organs and propagated as epithelial cyst-like structures *in vitro* with genomic, transcriptomic, and phenotypic properties resembling those of the original tissues [36]. Vlachogiannis et al. successfully developed PDOs from metastatic lesions of gastrointestinal primaries obtained at baseline and posttreatment time points [36]. Concordance of immunohistochemical markers and genetic aberrations such as *ERBB2* and *FGFR2* amplifications between PDOs and parental tissue was observed, in addition to a 96% overlap in mutational spectrums. Furthermore, it was demonstrated that PDOs could predict response to both targeted therapies and chemotherapy and mirrored the development of drug resistance in tandem with clinical outcomes. Similar findings have been reproduced in 66 PDAC PDOs cultured from both tissue obtained from surgical resections and fine needle biopsies [37]. Both these studies highlight that PDOs are robust preclinical models that can provide accurate representation of tumour histopathology and molecular heterogeneity by predicting treatment efficacy *in vitro* and are posed to be an invaluable tool in future drug development with possible clinical utility in prospective therapeutic selection.

Large-scale efforts to accrue molecular profiles representative of the patient population are essential to better our understanding of this complex disease and identify newer, rational therapeutic options. An example is the PRECISION-Panc platform in the United Kingdom, which serves as a portal protocol for patients with PDAC that offers patients full molecular characterisation of tumour tissue using upfront genomic sequencing with subsequent access to appropriate clinical trials alongside an integrated preclinical development programme [38]. An example of a clinical trial affiliated with the PRECISION-Panc platform is PRIMUS-001, which aims at comparing the efficacy of a novel platinum-containing regimen FOLFOX-A (5-FU, oxaliplatin, and nab-paclitaxel) against gemcitabine and nab-paclitaxel, with an incorporated translational research objective to further identify and characterise biomarkers of response to DNA-damaging agents. By allowing bidirectional translation between the laboratory and the clinic, platforms such as PRECISION-Panc represent an important opportunity to obtain and analyse multidimensional datasets which can enable identification of novel pathophysiology and treatment approaches to inform the direction of future research within the field.

## 4. Selected New Treatment Approaches on the Horizon

### 4.1. Targeting DNA Damage Repair Signalling

Endogenous and exogenous stressors such as reactive oxygen species and cytotoxic agents continuously produce DNA damage. This triggers a coordinated response orchestrated by an intricate network of DNA damage response (DDR) proteins to maintain genomic integrity and sustain cell viability. Consequently, defective DDR is associated with susceptibility to cancers including PDAC, ovarian, breast, and prostate cancers [39, 40]. The prevalence of a deleterious germline mutation in *BRCA1* and *BRCA2*, which encode for mediators of homologous recombination (HR), is estimated at 5% in PDAC patients [41], while aberrations in *PALB2* [42], *ATM* [43], *ATR* [44], *RAD51* [45], and *CHK1/2* [44] genes can also confer a HR-deficient or BRCA-like phenotype. Additionally, approximately 14% of PDACs have large numbers of structural variation events, resulting in genomic instability and DDR deficiency [31].

Impaired DDR signalling has classically been targeted by DNA-damaging agents such as platinums and alkylating agents. Agents interfering with DNA repair, most notably polyADP-ribose polymerase (PARP) inhibitors, are currently being assessed in PDAC. Clinical evaluation of PARP inhibitors in combination with chemotherapy (NCT01585805) is underway. Recently, results from the POLO study demonstrated that patients with germline BRCA-mutated metastatic PDAC who achieve disease control following a minimum of 16 weeks of platinum-based first-line chemotherapy reported a statistically significant improvement in PFS (7.4 months vs. 3.8 months, HR 0.53, 95% CI 0.35 to 0.82; *p* = 0.004) when randomised to receive olaparib over placebo. Although an OS benefit was not detected, these results provide a basis for the potential clinical application of a new class of therapeutics in a novel clinical setting within pancreatic cancer management [46]. Beyond BRCA mutant patients, HR-deficient patients represent another population who could derive benefit from agents that target DNA damage repair signalling.

### 4.2. Understanding the Desmoplastic Stroma and Immune Microenvironment in Pancreatic Ductal Adenocarcinoma

PDACs are encased by a desmoplastic reaction, which forms a fibrous layer of tissue surrounding malignant cells in both primary and metastatic lesions [47]. This consists of a diverse network of cellular components, including cancer-associated fibroblasts (CAFs), endothelial cells, immune cells, and extracellular matrix. The majority of these cells are CAFs, which secrete extracellular matrix proteins such as collagen, proteoglycans, fibronectin, matrix metalloproteases, and glycosaminoglycans [48]. While the resultant stroma is thought to be integral in promoting cancer growth and metastasis in addition to forming a physical barrier that facilitates a hypoxic environment and limits drug penetration [49], there is an increasing body of evidence to suggest that CAFs may restrain rather than promote PDAC tumour growth [50, 51]. Recent observations demonstrating significant intra- and intertumoural heterogeneity in patient-derived CAF primary cultures have provided insight into the complexity of CAFs and its role in the desmoplastic stroma [52] and support the hypothesis that CAFs play a context-dependent role in PDAC tumorigenesis and progression.

Although PDAC has a relatively low mutational load with a median somatic mutational prevalence of 1 mutation/megabase [53], the majority of PDACs express candidate neoantigens required to generate an antitumour response [54]. Additionally, it has been shown using *in silico* neoantigen prediction that tumours with the highest number of neoantigens alongside the most abundant CD8+ T-cell infiltrates are associated with the longest survival [55]. The presence of dendritic cells, a form of antigen-presenting cell required to stimulate a T-cell response, is infrequent in PDAC and tends to be immature even when present, compromising tumour antigen recognition and T-cell activation [56]. While PDAC tumour samples also demonstrate robust presence of tumour-infiltrating lymphocytes, active immune suppression mechanisms mediated by the presence of T regulatory cells, myeloid-derived suppressor cells (MDSCs), tumour-associated macrophages (TAMs), and inhibitory cytokines such as TGF-*β*, IL-10, and nitric oxide synthase mitigate against effective T-cell responses [54]. In light of these immunosuppressive mechanisms, PDAC is regarded as an immune-quiescent tumour.

The TME permits cross talk between malignant cells, its stromal components, and immune cells, creating a dynamic milieu which evolves throughout the course of the disease. Clearly, understanding the role of the desmoplastic stroma and its immune microenvironment is a crucial step in developing new therapeutic targets in PDAC, improve drug delivery, and broaden the clinical application of immunotherapy in this disease.

### 4.3. Targeting Components of the Desmoplastic Stroma

Interest was initially focussed on targeting the Hedgehog pathway, a key driver of the desmoplastic process. Its promise was first highlighted when the combination of saridegib, a Hedgehog inhibitor, and gemcitabine induced a transient increase in intratumoural vascular density and intratumoural levels of gemcitabine, resulting in improved survival in a patient-derived xenograft murine model [57]. These preclinical observations failed to translate into clinical benefit in early phase clinical studies. Further research has since shown that stromal constituents driven by the Hedgehog pathway act to restrain rather that promote tumorigenesis in PDAC [51, 58], highlighting the diverse roles of individual stromal components which subsequently pose challenges to successful drug development.

Focal adhesion kinases (FAKs) are nonreceptor tyrosine kinases that regulate cell signalling within the TME. They are frequently overexpressed in several advanced-stage solid tumours, including PDAC, and are important regulators of the fibrotic and immunosuppressive TME in PDAC when hyperactivated [59]. FAK inhibition has demonstrated antiproliferative therapy *in vitro* [60] and extended response to gemcitabine and nab-paclitaxel in patient-derived xenograft models [59]. These preclinical observations collectively form the basis of clinical trials incorporating FAK inhibition with trametinib (NCT02428270), gemcitabine (NCT02546531), and pembrolizumab (NCT02758587) (Tables 2 and 3). Preclinical models of solid tumours lacking neurofibromatosis type 2 (*NF2*) tumour suppressor gene product, merlin, exhibit exquisite sensitivity to FAK inhibition monotherapy [61], a finding that has also been recapitulated in early phase clinical trials [62]. Merlin deficiency is noted in over 40% of PDAC and is associated with adverse prognostic factors such as higher T stage, increased lymph node disease, and poorly differentiated histology [63]. The integration of predictive biomarkers of response similar to merlin loss could form the rationale for future biomarker-selected trials.

Another TME component that holds potential as a therapeutic target in PDAC is hyaluronan (HA), a glycosaminoglycan found in the stromal matrix. Structurally, HA has multiple anionic repeats that attract cations, resulting in osmotic swelling [64]. Tumours with high levels of HA have higher intratumoural interstitial gel fluid pressure which acts as a barrier to perfusion and reduces drug penetration to cancer cells [65]. When tested in preclinical models, pegylated recombinant human hyaluronidase (PEGPH20) was shown to degrade HA with subsequent normalisation of interstitial fluid pressures and reexpansion of the microvasculature within PDAC tumours [66]. Phase II evaluation has shown that PEGPH20 in combination with gemcitabine and nab-paclitaxel is particularly efficacious in patients with HA-high tumours, defined as extracellular matrix HA staining ≥ 50% of tumour surface at any intensity [67], and a phase III trial is ongoing in this subgroup of patients (NCT02715804). Interestingly, PEGPH20 given concurrently with FOLFIRINOX in an unselected group of patients resulted in detrimental OS outcomes and was associated with significantly higher rates of grade ≥ 3 toxicity [68]. These results highlight the importance of considered selection of individual combination therapy components as the interest in combinatorial strategies intensifies and becomes an increasingly common treatment approach in this tumour type. Efforts to exploit various targets within the desmoplastic stroma such as angiogenesis, hypoxic environment with the TME, and the Wnt-*β*-catenin pathway as therapeutic targets have also resulted in negative results to date (Table 2).

### 4.4. Extending the Impact of Immunotherapy in Pancreatic Ductal Adenocarcinoma

Although immune checkpoint inhibition has transformed the disease trajectory of tumour types historically associated with poor outcomes such as melanoma and non-small-cell lung cancer, these results have not been reproduced by both anti-CTLA-4 and anti-PD-L1 agents as monotherapy [69, 70] or in combination in biomarker-unselected populations with PDAC [71]. Immune checkpoint inhibition (ICPi) is only currently indicated in patients with mismatch repair- (MMR-) deficient disease, for which pembrolizumab received tumour-agnostic approval after it was shown to induce responses across multiple tumour types with this phenotype, including PDAC [72, 73]. The prevalence of MMR deficiency in PDAC is estimated between 1 and 2% [74], limiting the benefit of immune checkpoint inhibition to this small proportion of patients.

To that end, various combinatorial strategies aimed at priming the immune response in preparation for ICPi therapy have been explored in PDAC. For instance, chemotherapies facilitate dendritic cell recruitment and activation [75] in addition to tumour-specific antigen release [76], and studies evaluating the combination of anti-CTLA4 and anti-PD1/PD-L1 antibodies alongside chemotherapies are ongoing (Table 3).

Other combinations include agents targeting the desmoplastic stroma, such as PEGPH20 and FAK discussed previously. The C-X-C motif chemokine receptor type 4 (CXCR4)/stromal-derived factor-1 (CXCL12) is another candidate for combination with ICPi. Cancer stem cells with strong CXCR4 expression on the invasive front have been found to be a driver of metastatic behaviour and are prerequisite for the development of liver metastases [77]. CXCL12 secreted by CAFs has been shown to mediate immunosuppression, and its inhibition with AMD3100 induced rapid T-cell accumulation and synergistic activity with an anti-PD-L1 therapy in a PDAC mouse model [78]. Clinically, a phase II trial is underway with early results reporting that BL-8040 with pembrolizumab achieved a median OS of 7.5 months in patients receiving this as second-line therapy [79] (NCT02826486, Table 3).

As myeloid cells such as TAMs and MDSCs are important mediators of immune evasion, these have been identified as potential therapeutic targets in PDAC in the hope of overcoming its innate immunologically resistant phenotype. For example, C-C chemokine receptor type 2 (CCR2) and colony-stimulating factor-1 receptor (CSF1R) are involved in the recruitment and differentiation of TAMs within the PDAC TME. CSF1R or CCR2 inhibition has been shown to reduce the numbers of pancreatic tumour initiating cells and improve chemotherapy efficacy [80], and CSF1R inhibition upregulates PD-L1 and CTLA-4 checkpoint molecules in response to ICPi [81]. CSF1R inhibitors are currently being evaluated in combination with immunotherapy and chemotherapy (NCT02526017, NCT02777710) (Table 3).

Newer immune targets such as the CXCR2 axis are also being evaluated with ICPi. The primary role of the CXCR2 axis is to regulate neutrophil migration to the site of inflammation [82], and CXCR2 signalling at the tumour border is a poor prognostic indicator in human PDAC [83]. CXCR2 inhibition has been found to augment T-cell entry and increase sensitivity to ICPi therapy when used in combination in a mouse model [84], providing a rationale to pursue clinical testing (NCT02583477) (Table 3).

Another target thought to have immunomodulatory capabilities in PDAC is Bruton's tyrosine kinase (BTK), a Tec family nonreceptor tyrosine kinase that is required for B-cell receptor signalling. The BTK inhibitor, ibrutinib, has also been shown to exert antifibrotic effects on the desmoplastic stroma through inhibition of mast cell activity [85], as well as amplify cytotoxic T-cell activity with subsequent enhancement of responsiveness to chemotherapy in PDAC murine models [86]. Despite this encouraging preclinical data, a phase III trial randomising patients between gemcitabine/nab-paclitaxel in combination with ibrutinib or gemcitabine/nab-paclitaxel with placebo as first-line treatment of metastatic PDAC failed to meet its primary endpoints of PFS and OS (NCT02436668) [87] . Acalabrutinib is currently being assessed in combination with pembrolizumab at the phase 2 level, with preliminary results indicating an acceptable safety profile and encouraging antitumour activity in a pretreated PDAC patient population (NCT0236048) (Table 3).

Various types of vaccine therapies are also in active development. Personalised peptides designed to prevent progressive tolerance to cancer-related antigens given concurrently with gemcitabine have also shown promise [88] but require further investigation to establish its efficacy and development into a deliverable treatment modality. Another vaccine-based approach is whole-cell tumour vaccines, which enable multiple antigens to be targeted simultaneously to elicit a more robust T-cell response. Amongst the most studied is GVAX, which is genetically engineered to secrete GM-CSF, a cytokine that mobilises leucocytes to the TME and generates large immunoglobulin (Ig) G and IGM responses [89]. GVAX is thought to be an ideal primer for checkpoint inhibition, as it increases immunogenicity by inducing T-cell infiltration and formation of tertiary lymphoid aggregates [90], as well as upregulates the PD-1/PD-L1 pathway in PDAC patients [91]. Based on preclinical data suggesting that sequential administration of two vaccines to firstly “prime” the immune system then “boost” the immune response achieves synergistic enhancement of T-cell induction, the combination of GVAX and CRS-207, a live-attenuated *Listeria monocytogenes* vaccine expressing mesothelin, was evaluated at the phase II level. These results demonstrated extended survival of PDAC patients with minimal toxicity with this combination [92]. However, administration of CRS-207/GVAX or CRS-207 alone did not amount to a survival benefit over single-agent chemotherapy in a pretreated metastatic PDAC cohort (NCT02004262) [93]. Ongoing evaluation of its clinical potential in combination with ICPi therapy is currently being investigated (Table 3).

Additionally, the modulation of the gut microbiome in PDAC is an emerging field. The cancerous pancreas has been found to harbour distinct and more abundant gut microbiota in comparison to normal pancreatic tissue in both mice and humans [94]. Bacterial ablation using antibiotic therapy has been shown to be protective against oncogenesis, reversed intratumoural immune tolerance, and increased susceptibility to immune checkpoint blockade [94]. On this basis, the effects of metronidazole and ciprofloxacin given with pembrolizumab on immune activation in pancreatic cancer tissue in patients with surgically resectable PDAC will be investigated in an upcoming pilot study (NCT03891979).

A further immunotherapeutic approach is the application of tumour-oncolytic viruses (TOVs) to selectively infect, replicate in, and lyse tumour cells to unleash virions that can subsequently infect adjacent tumour cells, a process that potentiates an inflammatory response through immunogenic cell death [95]. Cell lysis also releases pathogen- and damage-associated molecular pattern molecules which activate the innate immune response, as well as viral- and tumour-associated antigens which stimulate the adaptive immune response [95]. Adenoviruses have been most extensively evaluated thus far. Given alone, ONYX-015, an adenovirus that selectively replicates and lyses in cells with p53 abnormalities, failed to induce objective responses in a phase I dose escalation study following intratumoural injection or exhibit evidence of viral replication [96] but resulted 2 partial responses and disease control in 8 patients when administered alongside gemcitabine in a cohort of 21 patients [97]. Phase II data investigating pelareorep, a reovirus, in combination with gemcitabine induced a partial response in one patient (*n* = 29) and PD-L1 upregulation following treatment [98], suggesting an immunomodulatory effect in PDAC and providing a rationale for pursuing combination therapy with immune checkpoint blockade. Pelareorep in combination with pembrolizumab and single-agent chemotherapy has since resulted in disease control in three of five evaluable patients in relapsed metastatic PDAC patients with a manageable safety profile (NCT02620423) (Table 3) [99].

Adoptive T-cell transfer techniques are also being investigated in the context of PDAC. Chimeric antigen receptor T-cell (CAR-T) therapy, which uses T-cells engineered to recognise a specific tumour antigen, appears to be the most effective method available. CAR-T therapy targeted to MUC1, a neoantigen expression in a variety of cancers including PDAC, has been shown to demonstrate target-specific cytotoxicity and improved survival in xenograft models of PDAC [100], with a phase I/II clinical study currently recruiting (NCT02587689). Other targets under clinical evaluation are mesothelin and carcinoembryonic antigen (NCT02465983, NCT02349724), the former having preliminary data suggesting activity in patients with PDAC [101].

## 5. Conclusion

The systemic treatment of advanced PDAC remains challenging. Although clinical trials have led to incremental gains in a survival benefit in recent years, significant improvements in patient outcomes have remained elusive for decades. Developing methods to improve the resolution of patient selection is crucial to exploit the benefit derived from existing therapies while we wait for newer therapies that target stromal elements or the TME to be fully evaluated and approved. Simultaneously, further translational and clinical work will be essential to advance our understanding of novel therapeutic targets and methods of manipulating the immune microenvironment to ensure the successful translation of rational precision therapeutics into the clinic. Finally, combinatorial strategies supported by sound biological rationale hold potential to fulfil the persistent unmet need posed by this aggressive malignancy.

## Figures and Tables

**Table 1:** Summary of first- and second-line trials in metastatic pancreatic ductal adenocarcinoma.

Reference	Treatment arms	*N*	Primary endpoint	Response rate (%)	Median PFS (months)	Median OS (months)
First-line therapy						
Burris et al. [8]	Gemcitabine vs.	126	CBR	23.8	2.1	5.65
	5-FU			4.8	1.2	4.41
				*p* = 0.0022	*p* = 0.0002	*p* = 0.0025
Cunningham et al. [14]	Gemcitabine-capecitabine vs.	533	OS	19.1	5.3	7.1
	gemcitabine			12.3	3.8	6.2
				*p* = 0.034	HR 0.78	HR 0.86
					*p* = 0.004	*p* = 0.08
PRODIGE4/ACCORD 11 [6]	FOLFIRINOX vs.	342	OS	31.6	6.4	11.1
	gemcitabine			9.4	3.3	6.8
				*p* < 0.001	HR 0.47	HR 0.57
					*p* < 0.001	*p* < 0.001
MPACT [15]	Gemcitabine/nab-paclitaxel vs.	861	OS	23	5.5	8.5
	gemcitabine			7	3.7	6.7
				*p* < 0.001	HR 0.69	HR 0.72
					*p* < 0.001	*p* < 0.001

Second-line therapy						
CONKO-003 [18]	OFF vs.	168	OS	NR	2.9	5.9
	FF				2.0	3.3
					HR 0.68	HR 0.66
					*p* = 0.019	*p* = 0.010
PANCREOX [19]	mFOLFOX6 vs.	108	PFS	13.2	3.1	6.1
	5-FU+leucovorin			8.5	2.9	9.9
				*p* = 0.361	HR 1.00	HR 1.78
					*p* = 0.99	*p* = 0.024
NAPOLI-1 [21]	Nal-IRI+5-FU/LV vs.	417	OS	17	3.1	6.2
	5-FU/LV			1	1.5	4.2
				*p* < 0.000	HR 0.57	HR 0.75
					*p* = 0.0001	*p* = 0.039
	Nal-IRI vs.			6	2.7	4.9
	5-FU/LV			1	1.6	4.2
				*p* = 0.02	HR 0.81	HR 1.07
					*p* = 0.81	*p* = 0.568

Abbreviations: CBR: clinical benefit response; FF: leucovorin+fluorouracil; HR: hazard ratio; FOLFIRINOX: 5-fluorouracil, irinotecan+oxaliplatin; mFOLFOX6 (modified FOLFOX6): 5-fluorouracil, leucovorin, and oxaliplatin; OFF: oxaliplatin+leucovorin+fluorouracil; LV: leucovorin; Nal-IRI: nanoliposomal irinotecan; OS: overall survival; PFS: progression-free survival; 5-FU: 5-fluorouracil.

**Table 2:** Selected clinical trials targeting desmoplastic stromal components in metastatic pancreatic ductal adenocarcinoma.

Target	Reference/trial number	Intervention	Phase	Status	*N*	Endpoints	Results
Hedgehog	Catenacci et al. [102] NCT01064622	Vismodegib+gemcitabine	Ib/II	Completed	Phase 1b: 7 Phase II: 106	PFS, OS, ORR, safety	(i) Phase Ib: no safety issues identified (ii) Phase II: no significant difference in PFS, OS, and ORR
	Ko et al. [103] NCT01383538	IPI-926+FOLFIRINOX	Ib	Completed	15	MTD, PFS, ORR, CA19-9 decline, safety	(i) MTD reached at 1 dose level below standard FOLFIRINOX (ii) trAEs: LFT abnormalities, neuropathy, nausea, vomiting, and diarrhoea (iii) ORR 67.7% (iv) CA19-9 decline in 66.7% when elevated at baseline
	Richards et al. [104]^∗^ NCT01130142	IPI-926+gemcitabine	Ib	Completed	16	Safety, MTD, OS, PK, PFS, TTP, ORR	(i) MTD IPI-926 160 mg OD (ii) No IPI-926-related serious AEs (ii) Median PFS >7 months

FAK	Aung et al. [105]^∗^ NCT02428270	GSK002256098+trametinib	II	Ongoing	16	ORR, safety, PFS	(i) PD as best tumour response in 10/11 and SD in 1/11 evaluable patients (ii) Median PFS 1.6 months (95% CI 1.5-1.8) (iii) Median OS 3.6 months (95% CI 2.7-not reached) (iv) No grade ≥ 3 trAEs
	NCT02546531 [106]	Defactinib+gemcitabine	I	Ongoing	N/A	RP2D, ORR, PFS, OS	N/A

Wnt-*β*-catenin	Ko et al. [107]^∗^ NCT01764477	PRI-724+gemcitabine	Ib	Completed	20	MTD, PK, pharmacodynamics, ORR	(i) MTD not reached (ii) Grade ≥ 3 trAEs: abdominal pain, neutropenia, anaemia, fatigue, and elevated ALP (iii) No DLTs (iv) SD as best response in 8 patients (40%) (v) Median PFS = 2.0 months
	Dotan et al. [108]^∗^ NCT02050178	Ipafricept (IPA)+gemcitabine/nab-paclitaxel	Ib	Completed	26	Safety, MTD, PK	(i) IPA-related AEs in ≥20% of patients: fatigue, nausea, vomiting, anorexia, and pyrexia (ii) IPA-related grade ≥ 3 AEs: AST elevation, nausea, maculopapular rash, vomiting, and WBC decrease (iii) No DLTs or fragility fractures (iv) PR in 9/26 evaluable patients (34.6%) (v) SD in 12/26 patients (46.2%) (vi) Study closed due to termination of the programme by sponsor (a) Median PFS 5.9 months (95% CI 3.4-18.4); median OS 9.7 months (95% CI: 7.0-14) at data cut-off

PEGPH20	Hingorani et al. [67] NCT01839487	PEGPH20+gemcitabine/nab-paclitaxel	II	Completed	279	PFS, ORR, OS, safety, PK	(i) Overall median PFS 6.0 months (PAG) vs. 5.3 months (AG) (HR 0.73; 95% CI, 0.53 to 1.00) (ii) In HA-high subgroup: (a) Median PFS 9.2 months (PAG) vs. 5.2 months (AG) (HR 0.51; 95% CI, 0.26 to 1.00; *p* = 0.048) (b) Median OS 11.5 vs. 8.5 months (HR 0.96; 95% CI, 0.57 to 1.61) (c) ORR 46% (PAG) vs. 34% (AG) (iii) No significant difference in TE events with LMWH prophylaxis (iv) Grade 3/4 trAEs: muscle spasms, neutropenia, myalgia
	NCT02715804 [109]	PEGPH20+gemcitabine/nab-paclitaxel	III	Ongoing	N/A	OS, PFS, ORR, DOR, safety	N/A
	Ramanathan et al. [68] NCT01959139	PEGPH20+mFOLFIRINOX	II	Completed	114	OS, PFS, ORR, safety	(i) HR 2.07 in favour of control arm at interim futility analysis (ii) Median OS mFOLFIRINOX vs. combination: 14.4 months (95% CI, 10.1 to 15.7 months) vs. 7.7 months (95% CI, 4.6 to 9.3 months) (iii) Grade 3/4 trAEs significantly higher in combination arm (OR 2.7; 95% CI, 1.1 to 7.1)

Hypoxia	Borad et al. [110] NCT01144455	TH302+gemcitabine	II	Completed	214	PFS, OS, ORR, change in CA19-9, PS, and pain score	(i) Median PFS combination vs. gemcitabine: 5.6 vs. 3.6 months (HR 0.61; 95% CI, 0.43 to 0.87; *p* = 0.005) (ii) TH302-related AEs: skin and mucosal toxicities and myelosuppression; unrelated to treatment discontinuation
	Van Custem et al. [111]^∗^ NCT01746979	TH302+gemcitabine	III	Completed	693	OS, PFS	(i) Median OS: 8.7 months (TH302+gemcitabine) vs. 7.6 months (gemcitabine+placebo) (HR = 0.84, 95% CI 0.71-1.01, *p* = 0.059) (ii) Median PFS: 5.5 months (TH302+gemcitabine) vs. 3.7 months (gemcitabine+placebo) (HR = 0.77, 95% CI: 0.65-0.92, *p* = 0.004) (iii) Similar safety profile between both arms

Angiogenesis	Kindler et al. [112]	Bevacizumab+gemcitabine	III	Completed	602	OS, PFS, ORR, safety	(i) No significant difference in median OS and PFS (ii) Grade 3/4 hypertension and proteinuria significantly higher in combination arm
	NCT03316599 [113]	INC280+AV-299+gemcitabine/nab-paclitaxel	I	Ongoing	N/A	MTD, ORR, PFS, safety	N/A

References marked with ^∗^ denote results derived from published abstracts. Abbreviations: AE: adverse event; AG: gemcitabine/nab-paclitaxel; CBR: clinical benefit response; CI: confidence interval; DLT: dose-limiting toxicities; DOR: duration of response; HA-high: hyaluronan-high; HR: hazard ratio; MTD: maximum tolerated dose; OR: odds ratio; ORR: overall response rate; OS: overall survival; PAG: PEGPH20+gemcitabine/nab-paclitaxel; PD: progressive disease; PFS: progression-free survival; PK: pharmacokinetics; PR: partial response; RP2D: recommended phase 2 dose; SD: stable disease; trAEs: treatment-related adverse events; TTP: time to tumour progression.

**Table 3:** Selected immunotherapy combination studies in metastatic pancreatic ductal adenocarcinoma.

Combination	Reference	Intervention	Phase	Status	*N*	Endpoints	Results
Chemotherapy + ICPi	Renouf et al. [114]^∗^ NCT02879318	Durvalumab+tremelimumab+gemcitabine/nab-paclitaxel vs. gemcitabine/nab-paclitaxel	II	Ongoing	11	OS, PFS, ORR, safety	(i) Data from safety run-in: (a) Grade ≥ 3 AEs: fatigue, myelosuppression, hyponatraemia, hypoalbuminaemia, deranged lipase, colitis (b) PR in 8/11 patients (73%) (c) Median DOR 7.4 months (d) Median PFS 7.9 months (95% CI 3.5-9.2 months)
	O'Reilly et al. [71]^∗^ NCT02558894	Durvalumab (D) vs. durvalumab+tremelimumab (D+T)	II	Completed	65	ORR, PFS, OS, BOR, DCR, PK (durvalumab+tremelimumab), presence of ADAs	(i) ORR 3.1% (D+T) vs. 0% (D) (ii) Median PFS 1.5 months (both arms) (iii) Median OS 3.1 (D+T) vs. 3.6 months (D) (iv) Grade ≥ 3 trAEs in D+T arm: diarrhoea, fatigue. Treatment discontinued in 3 patients
	Wainberg et al. [115]^∗^ NCT02309177	Nivolumab+gemcitabine/nab-paclitaxel	I	Completed	50	DLT, safety, PFS, OS, DCR, ORR, DOR	(i) Hepatitis as DLT, related to gemcitabine/nab-paclitaxel (ii) Grade ≥ 3 teAEs in 48/50 patients (96%) including anaemia, neutropenia, gastrointestinal events, hepatotoxicity, peripheral neuropathy, thrombocytopenia, and colitis (iii) 1 grade 5 teAE (respiratory failure) (iv) Median PFS 5.5 months (v) Median OS 9.9 months

PARPi+ICPi	NCT03851614	Olaparib+durvalumab	II	Ongoing	N/A	Genomic and immune biomarker changes, ORR, CBR, PFS, OS, safety	N/A

Desmoplastic stromal agent+ICPi	NCT02758587	Defactinib+pembrolizumab	I/II	Ongoing	N/A	Safety, ORR, DOR, PFS, change in FAK Y397 phosphorylation and immune cell infiltrate	N/A
	Wang-Gillam et al. [116]^∗^ NCT02546531	Defactinib+pembrolizumab+gemcitabine	I/II	Ongoing	17	RP2D, safety, ORR, PFS, OS	(i) Results from dose escalation cohort: (a) No DLTs (b) Level 5 dose as RP2D (defactinib 400 mg, pembrolizumab 200 mg, gemcitabine 1,000 mg/m^2^) (c) Common trAEs: fatigue, nausea, myalgia, vomiting, anorexia, pruritus, fever (d) SD in 7/13 evaluable patients (54%)
	Desai et al. [117]^∗^ NCT03193190	Atezolizumab+PEGPH20	Ib/II	Ongoing	N/A	ORR, safety, PFS, OS, DOR, PKs	N/A
	NCT03481920	Avelumab+PEGPH20	I	Ongoing	N/A	ORR, safety, OS, PFS, change in CA19-9	N/A
	Hidalgo et al. [79]^∗^ NCT02826486	Pembrolizumab+BL-8040	II	Ongoing	37	ORR, OS, PFS, DOR	(i) 29 evaluable patients (ii) PR in 1 patient (3.5%); SD in 10 patients (34.5%) (iii) Overall median OS 3.4 months; 7.5 months in second-line subgroup

Bruton's tyrosine kinase+ICPi	Overman et al. [118]^∗^ NCT02362048	Pembrolizumab+acalabrutinib vs. acalabrutinib	II	Ongoing	58	Safety	(i) No DLTs (ii) Grade ≥ 3 AEs in combination arm: dehydration, anaemia, hypotension (iii) Grade ≥ 3 AEs in monotherapy arm: anaemia, abdominal pain (iv) SD in 4/21 evaluable patients in monotherapy arm (v) PR in 3/23 and SD in 5/23 evaluable patients in combination arm

Myeloid inhibitor+ICPi	Wainberg et al. [119]^∗^ NCT02526017	Cabiralizumab+nivolumab	I	Ongoing	205	Safety, ORR, RD, PK, immunogenicity, biomarker analysis	(i) Grade ≥ 3 trAEs due to cabiralizumab in 43% of patients (ii) Treatment discontinuation due to AEs in 13% (iii) Most common grade ≥ 3 AEs: elevated CPK and AST. Reversible without sequalae (iv) 31 evaluable pretreated patients (a) 3 PR; 1 SD (b) ORR 10%
	NCT02777710	Pexidartinib+durvalumab	I	Ongoing	N/A	DLT, ORR, DOR, PFS, safety, PK	N/A

CXCR2 inhibitor+ICPi	NCT02583477	AZD5069+durvalumab	Ib/II	Completed	N/A	DLT, ORR, DOR, DCR, PFS, presence of ADAs, PK, safety	No results available

Oncolytic virus+ICPi	Mahalingam et al. [99]^∗^ NCT02620423	Pelareorep+pembrolizumab+chemotherapy (5-FU/leucovorin, gemcitabine, or irinotecan)	I	Completed	11	Safety, DLTs, ORR, PFS, immune markers	(i) Grade ≥ 3 teAEs in 8 patients (ii) PR in 1/5 and SD in 2/5 evaluable patients (iii) On-treatment biopsy showed reovirus infection and immune infiltrates

Vaccines+ICPi	Le et al. [120]^∗^ NCT02243371	GVAX/Cy+CRS-207±nivolumab	II	Ongoing	N/A	OS, safety, PFS, TTP, ORR, tumour marker kinetics	N/A
	NCT03190265	CRS-207 (±GVAX/Cy)+nivolumab+ipilimumab	II	Ongoing	N/A	ORR, safety, OS, PFS, DOR, TTP, tumour marker kinetics	N/A

References marked with ^∗^ denote results derived from published abstracts. Abbreviations: ADA: antidrug antibodies; AE: adverse event; AST: aspartate transaminase; BOR: best objective response; CBR: clinical benefit rate; CI: confidence interval; CPK: creatinine phosphokinase; DLT: dose-limiting toxicities; DCR: disease control rate; DOR: duration of response; ICPi: immune checkpoint inhibitor; ORR: overall response rate; OS: overall survival; PARPi: polyADP-ribose polymerase inhibitor; PK: pharmacokinetics; PR: partial response; PFS: progression-free survival; RP2D: recommended phase 2 dose; SD: stable disease; teAEs: treatment-emergent adverse events; trAEs: treatment-related adverse events; TTP: time to tumour progression.
